# Evaluation of Cadmium Bioaccumulation-Related Physiological Effects in *Salvinia biloba*: An Insight towards Its Use as Pollutant Bioindicator in Water Reservoirs

**DOI:** 10.3390/plants10122679

**Published:** 2021-12-06

**Authors:** Julia Emiliani, Wendi G. Llatance Oyarce, Lucas M. Salvatierra, Luís A. B. Novo, Leonardo M. Pérez

**Affiliations:** 1 Grupo de Biotecnología de Materiales y Medioambiente (Bio&TecMA), Instituto de Investigaciones en Ingeniería Ambiental, Química y Biotecnología Aplicada (INGEBIO-UCA), Facultad de Química e Ingeniería del Rosario, Pontificia Universidad Católica Argentina (UCA), Av. Pellegrini 3314, Rosario S2002QEO, Santa Fe, Argentina or jemiliani@cbm.csic.es (J.E.); lucas_salvatierra@uca.edu.ar (L.M.S.);; 2 Centro de Análisis Espectrofotométrico, Universidad Nacional de Jaén, Jirón Cuzco 250, Jaén 06801, Peru; wendi.llatance@unas.edu.pe; 3 National Council for Scientific and Technical Research (CONICET), Ministry of Science, Technology and Productive Innovation, Godoy Cruz 2290, Buenos Aires C1425FQB, Argentina; 4 Scotland’s Rural College, West Mains Road, The King’s Buildings, Edinburgh EH9 3JG, UK

**Keywords:** metal-polluted waters, cadmium, biomonitoring, autochthonous macrophyte species, *Salvinia biloba*

## Abstract

Free-living macrophytes play an important role in the health of aquatic ecosystems. Therefore, the use of aquatic plants as metal biomonitors may be a suitable tool for the management of freshwater reservoirs. Hence, in this study, we assessed the effects of cadmium (Cd) in *Salvinia biloba* specimens collected from the Middle Paraná River during a 10-day experiment employing artificially contaminated water (100 μM Cd). *S. biloba* demonstrated a great ability for Cd bioaccumulation in both the root-like modified fronds (named “roots”) and the aerial leaf-like fronds (named “leaves”) of the plants. Additionally, Cd toxicity was determined by the quantification of photosynthetic pigments (chlorophylls *a* and *b*, and carotenoids), flavonoids, and soluble carbohydrate contents in *S. biloba* over time (1, 3, 5, 7, and 10 days). In general, deterioration was more pronounced in leaves than in roots, suggesting a greater implication of the former in long-term Cd sequestration in *S. biloba*. Deleterious effects in the appraised parameters were well correlated with the total amount of Cd accumulated in the leaves, and with the qualitative changes observed in the plants’ phenotype during the 10-day metal exposure assay. The flavonoids and carotenoids in leaves were highly affected by low Cd levels followed by root carbohydrates. In contrast, chlorophylls and root flavonoids were the least impacted physiological parameters. Therefore, our results demonstrate that *S. biloba* displays dissimilar organ-linked physiological responses to counteract Cd phytotoxicity and that these responses are also time-dependent. Though further research is needed, our work suggests that easy-handled physiological data obtained from autochthonous free-floating *S. biloba* specimens may be used as a valuable tool for metal-polluted water biomonitoring.

## 1. Introduction

Free-floating macrophytes (i.e., hydrophytes) are macroscopic aquatic plants that play an important role in aquatic ecosystem health. These organisms are key components in natural wetlands and can serve as bioindicators of a variety of ecosystem stressors such as nutrient runoff, changes in hydrologic regime, and exotic species invasion. In addition, macrophytes may be also indicative of water contamination caused by human activities such as industrial, agricultural, and mining exploitation [1,2,3,4]. Therefore, the use of macrophytes as biomonitors of stream health should be considered as a useful tool for the management of aquatic ecosystems. However, bioindicators estimating the pollutants affecting the biota in an ecosystem are often purely qualitative in nature.

During the last two decades, free-floating macrophytes have been extensively studied in relation to its growth, metabolic response, and phytoremediation capacity towards several environmental contaminants, especially heavy metals [5,6,7,8,9,10,11]. Moreover, the phytotoxic effects of metal contaminants on aquatic vegetation have been addressed with regard to changes in plant phenotype and population, and/or the impact on different physiological parameters due to metal stress (e.g., photosynthetic pigment content, antioxidant enzyme activities, etc.). In fact, when growing in contaminated sites, macrophytes may develop differential phytopathological alterations in different organs (i.e., submerged roots and aerial leaves) showing a high morphological and metabolic plasticity [12,13,14,15,16,17,18,19]. Hence, in macrophyte ecotoxicological studies, it seems appropriate to evaluate metal-induced changes on different biological parameters in both plant organs since these results may differ and, in this way, new insights for their use as pollutant bioindicators could be provided. However, limited information is available in the scientific literature about the use of macrophytes as biomonitoring agents for heavy metal-polluted aquatic environments from a physiological perspective [4,5,17]. On the other hand, the use of aquatic plants for phytoremediation of metal-polluted water and wastewater has been comprehensively demonstrated as a highly efficient and cost-effective method for toxic metal elimination [5,6,7,8,9,10,11,12,13,14,15,16,17,18,19]. Thus, local macrophyte species with an elevated biomass production rate enable more sustainable and economically viable phytoremediation strategies, especially in areas where communities have limited financial resources [12,13,19].

In Argentina, the ecosystem surrounding the Middle Paraná River is well characterized by an extensive wetland area with copious and diverse aquatic vegetation due to the temperature and light favorable regime of this region [20,21]. Different macrophytes belonging to the *Salvinia* family obtained from Paraná River floodplain have been studied for metal removal from polluted environments. In particular, *Salvinia biloba* has showed a high growth rate, great capacity to survive under adverse environmental conditions, and proper heavy metal removal capacity, including cadmium (Cd), lead (Pb), copper (Cu), zinc (Zn), nickel (Ni), and chromium (Cr) [6,12,13,18,19].

Globally, Cd is one of the most concerning water pollutants. Although its toxicity is a highly researched topic, Cd pollution is far from being under control [22,23]. Common sources of Cd include wastes from mining and metallurgical operations, electroplating industries, Cd-based batteries, and runoff water from agriculture soils impacted with Cd-containing fertilizers, among others. In addition, a large amount of Cd is naturally released into rivers through the weathering of rocks [22]. Cadmium is highly toxic to the aquatic ecosystem, including fish, plants, and other organisms [24,25]. Moreover, long-term human exposure to Cd has been associated with kidney and chronic liver dysfunction and with an increased risk of prostate, lung, endometrium, bladder, and breast cancer [22,23,26]. In fact, this element is classified as a Group 1 human carcinogen, and it is also currently listed by the World Health Organization (WHO) as 1 of the 10 chemicals of major public concern [27]. Thus, Cd is considered a priority water pollutant from a monitoring perspective by most countries and international organizations.

To the best of our knowledge, there are no studies describing the biomonitoring ability of native *S. biloba* specimens exposed to Cd. Furthermore, there is limited information on the time-dependent effects of Cd on *S. biloba* physiological parameters [12]. Therefore, the aim of the present study was to evaluate the metal bioaccumulation capacity of autochthonous free-floating *S. biloba* specimens exposed to Cd-polluted water over a 10-day period. Additionally, chlorophylls (*a* and *b*), carotenoids, flavonoids, and soluble carbohydrate contents were also assessed in order to link easy-acquired physiological information with the potential role of these locally available macrophytes as biomonitoring agents for Cd water pollution.

## 2. Materials and Methods

### 2.1. Plant Collection and Characterization

Naturally-occurring *S. biloba* specimens were collected from an uncontaminated shallow lake located in a floodplain at the Middle Paraná River in front of Rosario city (32°52′35″ S; 60°40′33″ W, Santa Fe, Argentina), a natural wetland environment in which *S. biloba* develops conspicuously [12,13,20]. During manually collection, the plants were stored at ambient temperature in plastic recipients containing river water until they were transported to the laboratory. Once at the lab, the macrophytes were acclimated during 15 days at controlled room temperature (24 ± 2 °C) under natural light in a hydroponic system (20 L glass aquaria) containing a combination (50:50) of tap water and river water. Taxonomic classification of the collected macrophytes was performed based on morphological characteristics [13].

### 2.2. Cd Bioaccumulation Studies

Plants with uniform size and without any visual signs of deterioration (i.e., loss of turgor, defined chlorosis and/or necrosis areas on plant leaves, etc.) were chosen for further experimental purposes. The selected *S. biloba* specimens were placed (10.0 g wet basis) in beaker glass (600 mL) containing 400 mL of 100 µM Cd solution (i.e., 11.2 mgCd/L), prepared by diluting a commercial standard Cd solution (1000 mg/L, SCP Science, Quebec, Canada) in deionized water. The experiments were carried out at 24 ± 2 °C under artificial light (Osram Dulux L HE, München, Germany) with dark/light cycles of 12 h [12,13,20]. The pH of the solution (pH~6.0–7.0) was assessed during all the assays using an AD1030 digital pH-meter (Adwa, Nușfalău, Romania) in order to avoid possible Cd(OH)_2_ precipitation. Three experimental units (*n* = 3) were used as replicates for each exposure time (0, 1, 3, 5, 7, and 10 days) and the data were reported as mean value ± standard error (S.E.). At the end of each exposure time, total biomass was collected and gently washed with deionized water. Later, plant biomass was washed with 400 mL of 1.7 mM EDTA solution (concentration equivalent to a molar ratio EDTA/Cd ≥ 17) for 60 min under gentle orbital agitation (80 rpm) to eliminate Cd adsorbed on the plant surface. Then, the EDTA-washed biomass was rinsed with deionized water and separated in submerged root-like modified fronds (named “roots”), and aerial leaf-like fronds (named “leaves”), for further physiological parameter analysis (see Section 2.3). Subsequently, another portion of the washed biomass was dried at 90 °C to constant weight. Finally, 50.0 mg of dried biomass were treated with 1.0 mL 65% HNO_3_ analytical grade (Cicarelli, Santa Fe, Argentina) and heated at 120 °C in a digestion system for 2 h. Aliquots of digested samples were accordingly diluted with acidic water (0.15% *v/v* HNO3, Cicarelli, San Lorenzo, Argentina) in glass calibrated containers to quantify the amount of Cd accumulated in plant tissues by atomic absorption using a Varian AA240FS spectrophotometer (Varian Inc., Palo Alto, CA, USA). For calibration purposes, standard solutions of Cd (0.30, 0.60, 1.0, 2.0, 2.5, and 3.0 mg/L) were prepared by diluting a commercial standard solution (1000 mgCd/L, SCP Science, Quebec, Canada) with the necessary volume of acidified water (0.15% *v/v* HNO_3_; Cicarelli, San Lorenzo, Argentina) in glass calibrated containers, as recommended by the Varian AA240FS operational manual. All calibration procedures showed reproducible linear relationships (R^2^ > 0.98).

### 2.3. Physiological Parameters

#### 2.3.1. Quantification of Photosynthetic Pigments

Photosynthetic pigments (chlorophylls and carotenoids) were quantified from 50.0 mg (*n* = 3) of fresh biomass (FW) added with 96% (*v*/*v*) ethanol (1.0 mL) and incubated for 24 h in darkness at 4 °C. The extract was centrifuged (10 min, 4500× *g*) and the absorbance of the supernatant was measured at 480, 649, and 665 nm by using a Lambda 25 UV-vis spectrophotometer (Perkin Elmer, Boston, MA, USA). Contents of chlorophylls (*a* and *b*) and carotenoids (carotenes and xanthophylls) were expressed as µg/g (FW) and calculated according to [28]. Data were reported as the mean value ± standard error (S.E.) from three biological replicates (*n* = 3).

#### 2.3.2. Flavonoid Determination

The content of flavonoids was assessed as described in [15]. Briefly, 50.0 mg (*n* = 3) of fresh biomass (FW) were extracted for 8 h with 0.6 mL of acidic methanol (1% *v/v* HCl in methanol), followed by a second extraction with 1.2 mL of chloroform and 0.6 mL of distilled water. After vortexing, the extracts were centrifuged (5 min, 4500× *g*) and the absorbance of the supernatant was measured at 330 nm using a UV-vis Lambda 25 spectrophotometer (Perkin Elmer, Boston, MA, USA). Finally, the flavonoid content was expressed as absorbance units (A_330_) per gram of FW [12,13,15,16,17]. Data were reported as the mean value ± standard error (S.E.) from three biological replicates (*n* = 3).

#### 2.3.3. Soluble Carbohydrates

The content of soluble carbohydrates was determined according to [29]. Briefly, 100 mg (*n* = 3) of fresh biomass (FW) were mixed with 80% (*v*/*v*) methanol (2.0 mL) and heated (70 °C, 30 min). After being cooled, 1.0 mL of the extract was added with 6.0 mL of an acidic solution of 5% (*v*/*v*) phenol (Sigma-Aldrich, St. Louis, MO, USA). Finally, the mixtures were incubated at room temperature for 60 min, and the absorbance of the solution was measured at 490 nm using a UV-vis Lambda 25 spectrophotometer (Perkin Elmer, Boston, MA, USA) [15]. The concentration of soluble carbohydrates was expressed as mg/g FW using Glucose (50 mg) as the standard (Sigma-Aldrich, St. Louis, MO, USA) [13]. Data were reported as the mean value ± standard error (S.E.) from three biological replicates (*n* = 3).

### 2.4. Statistical Analysis

Statistical analyses were performed using the SigmaStat 3.5 program (Systat Software Inc., San Jose, CA, USA). Following the assessment of data normality and homogeneity of variances, the ANOVA test was used to compare the collected data between control and Cd-treated samples during the 10-day assay. Tukey’s *post-hoc* test was applied when the differences in the measured values were different (*p* < 0.05). Pearson correlation coefficients were computed in the R ver. 4.1.0 (R Foundation for Statistical Computing, Vienna, Austria) environment [30] to appraise the relationship between parameters.

## 3. Results and Discussion

### 3.1. Cd Bioaccumulation

Metal phytoremediation in *S. biloba* can be explained by a combination of several coordinated mechanisms involving the metal union to the plant biomass, the metal translocation through the cell wall, and finally the metal sequestration, transport, and storage into different cell compartments. In a recent work, we have demonstrated that Cd uptake within *S. biloba* biomass was the main removal mechanism used by these macrophytes to remove the metal from the water column [12]. In this study, we evaluate the pattern of Cd bioaccumulation in the root-like modified fronds (named “roots”) and the aerial leaf-like fronds (named “leaves”) of naturally occurring *S. biloba* specimens during 10 days of plant exposure to water artificially contaminated with 100 μM Cd.

**Figure 1:**
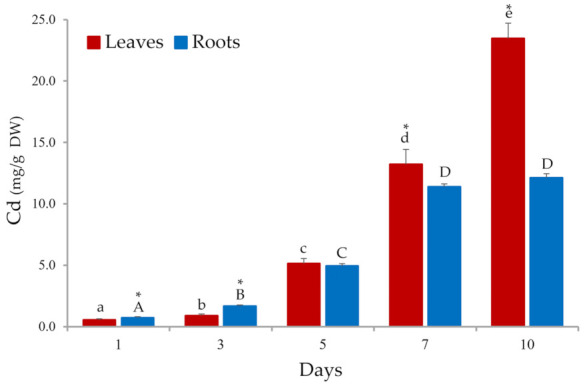
Metal bioaccumulation in roots and leaves of *S. biloba* specimens exposed to water contaminated with 100 µM Cd for 10 days. Different letters represent significant differences (*p* < 0.05) between Cd accumulated in roots (uppercase letters) or leaves (lowercase letters) at increasing exposure time. * Indicates significant differences (*p* < 0.05) between Cd accumulated in roots and leaves of *S. biloba* at the same exposure time. Data are reported as the mean value ± standard error (S.E.) from three biological replicates (*n* = 3).

Figure 1 shows the mean value of metal bioaccumulated (mg/g) in separate roots and leaves of Cd-treated plants after 1, 3, 5, 7, and 10 days of metal exposure for three biological replicates. As can be seen, Cd bioaccumulation in both organs was positively affected by the exposure time increase (Figure 1). During the first 72 h, *S. biloba* showed a greater capacity (*p* < 0.05) to incorporate Cd within plant roots (from 720 ± 76 μg/g at 24 h to 1650 ± 112 μg/g at 72 h) regarding the amount of the metal incorporated into the leaves (from 531 ± 118 μg/g at 24 h to 891 ± 131 μg/g at 72 h).

This behavior is in agreement with our previous report, proving that *S. biloba* uses different mechanisms for divalent cation removal from polluted waters [12]; where the fastest component of Cd uptake by these plants is surface adsorption followed by metal bioaccumulation in the submerged root-like modified fronds. This phenomenon occurs through the combination of physicochemical and biological processes including ionic bonds, chemical chelation, and cationic exchange during the adsorption phase, followed by a translocation step involving the action of transmembrane proteins and ionic channels [6,12,18,19,31,32]. Estrella-Gómez et al. [33] demonstrated the relationship between metal bioaccumulation and the activation of metal sequestration mechanisms mediated by phytochelatins in the roots of *Salvinia* minima exposed to Pb. Metal sequestration by cysteine- and glutamic acid-rich peptides such as glutathione, phytochelatins, and metallothioneins are some of the best well-known mechanisms of metal detoxification in plants. These proteins have the ability to bind toxic metals, forming complexes that are later stored in vacuoles and chloroplasts, thus reducing the deleterious effect of toxic metals to cells [34,35]. Hence, it may be expected that most *Salvinia* species share similar chelating mechanisms involving phytochelatin and/or metallothionein expression during Cd uptake [36,37]. In addition, Olguín et al. [32] have suggested that calcium channels may also be involved in the intracellular accumulation of divalent metals in *Salvinia* species. However, more evidence is needed to support this hypothesis.

Metal translocation to the aerial parts of the plant (i.e., leaves) is a much slower phase of the Cd removal mechanism used by *S. biloba*. During the first three days of metal exposure, the amount of Cd incorporated in plant leaves was significantly (*p* < 0.05) lower (891 ± 131 μg/g) than the metal concentration found in plant roots (1650 ± 112 μg/g). However, from day 5 onwards, the amount of Cd bioaccumulated in *S. biloba* leaves was highly increased (Figure 1), doubling the amount of the metal accumulated by the plant roots at the end of the assay (23,450 ± 1250 μg/g in leaves vs. 12,100 ± 340 μg/g in roots). This observation matches our previous results using autochthonous free-floating *S. biloba* specimens exposure to water contaminated with 100 μM Pb [38]. In such work, we demonstrated that after 30 days of metal exposure the amount of Pb accumulated in *S. biloba* biomass increased with the concomitant decrease in the total amount of Pb adsorbed onto plant roots. The finding suggests that this behavior involves the metal translocation/transport from roots into leaves by the presence of both transmembrane and intracellular metal-carrier proteins as part of a metal-tolerance mechanism developed by the plants [39].

The bioconcentration factor (BCF) can be employed as a gauge for metal-uptake efficiency in different macrophytes commonly used in phytoremediation trials [19]. In general, high BCF values denote high metal cumulative loads in the plant biomass for different metals, which can be desired for vegetal species selection in terms of designing a bio-based system for metal-polluted water treatment. Interestingly, as it can be inferred from Figure 1, BCF values significantly increase with exposure time in both plant organs, suggesting a high Cd cumulative capacity for *S. biloba*. However, the different physiological needs of a plant, the metal toxicity and its uptake kinetics could directly or indirectly affect the accumulative bioprocesses for a particular metal ion in different macrophytes species.

### 3.2. Phenotypic Evaluation of Cd Phytotoxicity in S. biloba

Cadmium is a non-essential element that negatively affects plant growth and development. It has been reported that aquatic plants belonging to *Salvinia* sp. and exposed to toxic metals show typical symptoms, such as leaf chlorosis/necrosis, the appearance of a brownish-red coloration on the leaf surface, or a reduced total biomass yield [6,12,13,17]. Moreover, a possible reason for Cd toxicity is its chemical similarity to ferrous ion (Fe^2+^), which can be replaced in Cd-exposed plants, thus affecting many vital physiological processes and producing several morphological and structural damages [17].

**Figure 2:**
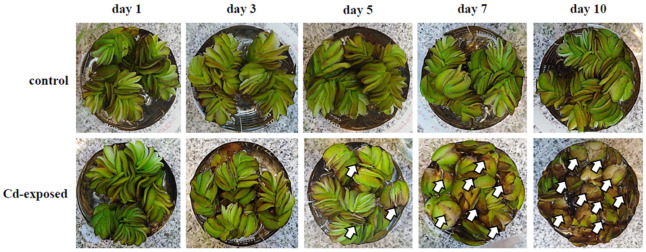
Representative photographs showing phenotypic alterations observed in *S. biloba* leaves during 10-day exposure to water contaminated with 100 μM Cd compared to the leaves of control plants. Arrows indicate some of the representative changes in leaves (chlorosis and signs of necrosis) described in Section 3.2.

In the present study, the phytotoxic effects observed in the biomass of naturally occurring *S. biloba* specimens over 10 days of metal exposure to water contaminated with 100 μM Cd are shown in Figure 2. A gradual response of *S. biloba* to Cd phytotoxicity was observed, mostly evidenced by a marked leaf chlorosis (i.e., green chlorophylls change towards darker areas), alterations in the size and the shape of the leaves, and the emergence of signs of necrosis (cell death) in both the juvenile fronds and in the most developed ones. In fact, the occurrence of the necrotized areas in the leaf surface of the treated ferns increased with time, and with the increase in the total amount of metal (μg/g DW) accumulated in such organs (Figure 1). This behavior was most pronounced from day 5 of metal exposure onwards in agreement with a related study by Wolff et al. [17]. Additionally, the necrotized areas presented dark brown pigmentation from the edge of the leaf toward the middle. At day 10, clear images of an opaque deposition along the middle lamellae of the plant biomass were detected, in conjunction with an entire leaf opening. Moreover, marked tissue necrosis in those areas of greatest contact with the Cd-contaminated water was spotted via stereomicroscopic analysis. This pattern could be associated with a reduced stomatal opening and a concomitant lowered rate of photosynthesis, and also with a diminished nutrient absorption and transportation [13,17]. On the contrary, the Cd-unexposed control plants showed no apparent changes in leaf phenotype after 10 days with respect to the initial condition (Figure 2), suggesting a proper conservation of its initial physiological state.

According to our observations, *S. biloba* specimens exposed to Cd presented morphological changes proportional to the concentration of metal bioaccumulated in the leaves. Therefore, these results support the potential use of this aquatic fern as ecological indicators of Cd presence in aquatic environments since phenotypic changes in plant leaves induced by the metal were clearly visible and easily quantified.

### 3.3. Cd Phytotoxicity in S. biloba

#### 3.3.1. Photosynthetic Pigments

The variation in the content of the photosynthetic pigments (i.e., chlorophylls and carotenoids) is frequently used to assess heavy metal toxicity in plants, including aquatic macrophytes [12,13,15]. Chlorophylls are the main chloroplast pigments responsible for collecting solar radiation during the photosynthetic processes [40]. Therefore, it is important to record changes in both major types of green pigment, chlorophyll *a* (chl *a*) and chlorophyll *b* (chl *b*). This is due to the fact that different metals could affect each chlorophyll component at different levels [12,13,14,15]. On the other hand, carotenoids are essential to photosynthesis acting as secondary pigments, pro-vitamin factors, and to eliminate reactive oxygen species (ROS) in the damaged tissues [40]. Considering that the content of photosynthetic pigments in floating fronds of *Salvinia* sp. is at least two to three times higher than that of the submerged roots [41,42], we restricted the evaluation of chl *a*, chl *b*, and carotenoid concentrations to the leaves.

**Figure 3:**
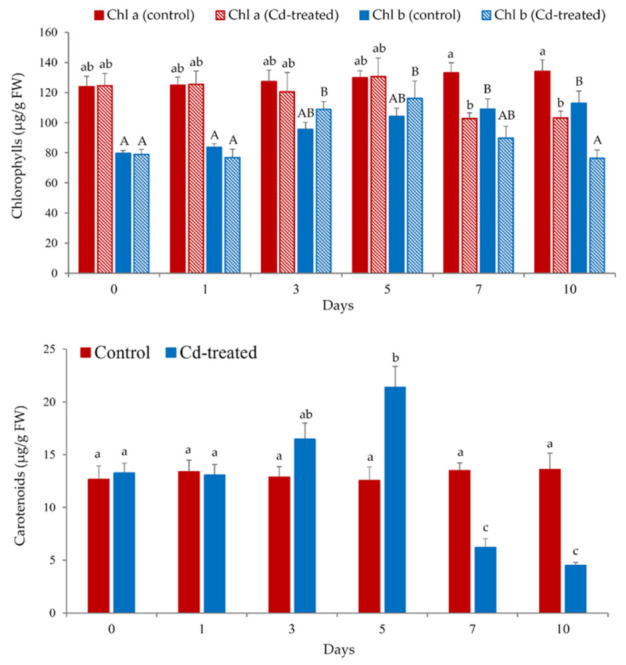
Photosynthetic pigment (chlorophylls, top; and carotenoids, bottom) content in *S. biloba* leaves during 10-day exposure to water contaminated with 100 μM Cd. Different letters (lowercase or uppercase) represent statistically significant differences (*p* < 0.05), e.g., “a” and “b” are statistically different from each other but not from “ab”. Data are reported as the mean value ± standard error (S.E.) from three biological replicates (*n* = 3).

As observed in Figure 3, chlorophylls were less affected by the metal exposure (100 μM Cd) than carotenoids. The concentration of chl *a* in the leaves of Cd-treated plants only significantly decreased (*p* < 0.05) at days 7 and 10 of metal exposure with respect to the control group. This result is consistent with the photographs depicted in Figure 2, in which plants exposed to Cd-contaminated water showed a marked increase in leaf chlorosis from day 7 onwards; whereas before that point the floating fronds exhibited a darker uniform green color likely related to greater chl *a* content.

Noticeably, chl *b* was more resistant to Cd accumulation in plant leaves, most likely due to a faster hydrolysis ratio of chl *a* compared with chl *b* when plants are under metallic stress [12,13]. Chl *b* is found in light harvesting complexes, playing a critical role in the absorption of light at 425–475 nm, where chl *a* absorbs significantly less than chl *b* [40]. Despite this, no plant showed reduction in biomass yield compared with the control group after a 10-day Cd exposure (Figure 2).

Carotenoid concentration in Cd-treated *S. biloba* specimens showed a significant (*p* < 0.05) increase at day 5, followed by a pronounced decrease at days 7 to 10 of metal exposure, in relation to the control group. Moderate concentrations of Cd caused an increase in carotenoids, which protects against stress, since these pigments are quenchers of ROS (^1^O_2_) and of triplet excited states of chlorophyll [40], reducing lipid peroxidation and consequent oxidative damage. However, the increasing accumulation of Cd significantly accelerates the degradation of the photosynthetic pigments in *S. biloba*. The reduction of these pigments most likely relates to the increase in ROS acquired from the damage of the photosystems. The ionic imbalance caused by excessive Cd may affect aminolevulinic acid synthesis (precursor of chlorophyll), increasing degradation of the pigments [15,43].

Oxidative stress is a well-known physiological effect of heavy metals in plants and other autotrophic aquatic organisms [15,43,44]. ROS are produced in the chloroplast, either as byproducts of O_2_ reduction or as a result of the presence of highly energized photopigments [45,46]. The observed increase in carotenoids (Figure 3) may be explained by the fact that xanthophylls and carotenes quench excess excitation energy to protect chlorophylls from oxidative damage and to stabilize the lipid bilayer of cell membranes, preventing ROS-induced lipid peroxidation [15,17,39,44,45]. However, metal excess in plant tissues can affect photosynthesis by a series of factors, including degradation and/or biosynthesis inhibition of chlorophylls, plastoquinone, and carotenoids; disruption of chloroplast organization; and changes in the composition of the thylakoid membrane where the photosynthetic pigments are deposited [15,17,39,43,45,46]. All of these processes can explain the decrease in photosynthetic pigments in *S. biloba* leaves from day 7 onwards, when the concentration of Cd in plant tissue exceeded 10.0 µg/g (dry basis) (Figure 1 and Figure 3).

In a recent study, we have investigated the effect of different metals (Cd, Cu, Pb, and Zn) on the photosynthetic activity of *S. biloba*. However, no changes in chlorophyll and carotenoid levels in the leaves of *S. biloba* were observed after 48 h of plant exposure to 50 and 100 μM Cd [12]. Thus, the results of this work highlight the significance of carrying out longer metal exposure trials to identify additional physiological changes that could operate as biomarkers of metal toxicity and as a tool for the selection of macrophytes for biomonitoring and remediation purposes.

#### 3.3.2. Flavonoid Content

**Figure 4:**
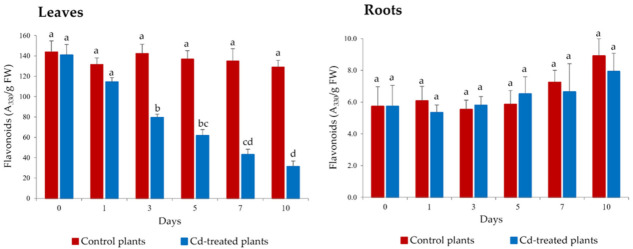
Flavonoid content in *S. biloba* leaves (left) and roots (right) during 10-day exposure to water contaminated with 100 μM Cd. Different letters represent statistically significant differences (*p* < 0.05), e.g., “c” is statistically different from “b” and “d”, but not from “bc” or “cd”. Data are reported as the mean value ± standard error (S.E.) from three biological replicates (*n* = 3).

The concentration of flavonoids in floating fronds of *S. biloba* exposed during 10 days to water containing 100 μM Cd was significantly (*p* < 0.05) impacted from the third day of metal challenge onwards (Figure 4).

Phenolic compounds are essential for the plant defense against stress caused by both biotic and abiotic agents [45]. In fact, flavonoids have been reported to be involved in metal tolerance for several *Salvinia* species, acting as metal ion-chelating agents and preventing the generation of metal-induced ROS. For example, Bizzo et al. [15] reported an increase in the amount of total phenolic compounds in *Salvinia auriculata* exposed for 48 h to 0.01, 0.1, 1.0, and 10 mM Cu(II). More recently, Prado et al. [14] observed an increase in the total amount of soluble thiols in leaves of *Salvinia rotundifolia* and *S. minima* after 7-day treatment with water contaminated with 20 mg/L Cr(VI) (i.e., ~385 μM).

In the present study, no increase in flavonoid content in *S. biloba* leaves was detected during the first 24 h of metal exposure. This result is in agreement with our previous report [12], possibly because the amount of Cd accumulated in the plant after this period was not detrimental at the oxidative level. However, prolonged exposure to Cd and its increasing accumulation in the leaves led to a significant decrease in the amount of flavonoids (*p* < 0.05) from day 3 onwards, suggesting that leaves of Cd-exposed *S. biloba* accumulated a metal concentration that was harmful to cell homeostasis and caused metabolic breakdown. Inversely, the concentration of flavonoids in *S. biloba* roots of control and Cd-treated specimens did not change during the entire trial (Figure 4).

It is worth noting that root flavonoids play significant roles in protecting the plants against pests and diseases, regulating root growth and functions, influencing different aspects of nitrogen cycle, and exerting allelopathic growth effects [47]. In addition, Fini et al. [48] suggested that in plants subjected to severe/prolonged stress, the very conditions that lead to the inactivation of antioxidant enzymes can also upregulate the biosynthesis of antioxidant flavonoids as a secondary ROS-scavenging system.

Different studies have been conducted in order to understand the molecular basis of the response of *Salvinia* sp. when exposed to metals. For example, Estrella-Gómez et al. [33] demonstrated the relationship between Pb accumulation and the activation of chelation and metal sequestration mechanisms mediated by phytochelatins in roots of *S. minima*. Moreover, these authors also analyzed the connection between Pb accumulation and changes in glutathione (GSH) levels in *S. minima* roots, suggesting that an increase in GSH biosynthesis may play an important role in protecting plant organs from the oxidative damage caused by Pb [49]. More recently, Leal-Alvarado et al. [50] analyzed the expression levels of genes coding for ATP-dependent tonoplast transporters (ABC transporters) involved in *S. minima* roots tolerance to Pb. The authors found a sharp increase in the expression of the above-mentioned genes in *S. minima* exposed to 40 μM Pb for 24 h, suggesting their participation in the control of root cell homeostasis. Although these data were the first attempt at understanding the molecular basis of *S. minima* tolerance to heavy metals, it is expected that other aquatic ferns belonging to the *Salvinia* genus share similar mechanisms to counteract the imbalance caused by toxic metal accumulation. Therefore, the reaction of *S. biloba a*gainst Cd stress is expected to be controlled by a complex and highly interrelated network of molecular and physiological approaches that help to counteract metal phytotoxicity [51].

#### 3.3.3. Soluble Carbohydrates

**Figure 5:**
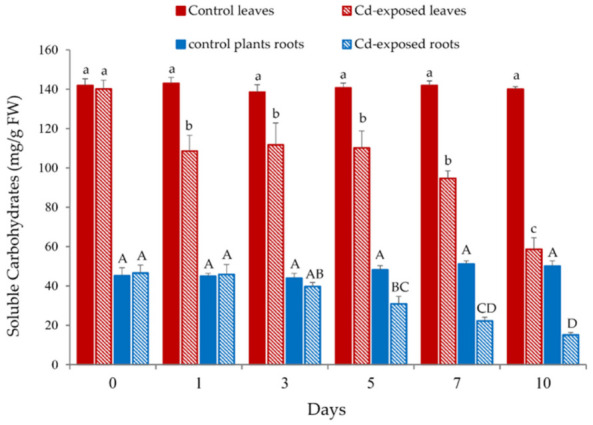
Soluble carbohydrate content in *S. biloba* leaves and roots during 10-day exposure to water contaminated with 100 μM Cd. Different letters (lowercase or uppercase) represent statistically significant differences (*p* < 0.05), e.g., “A” and “B” are statistically different from each other but not from “AB”. Data are reported as the mean value ± standard error (S.E.) from three biological replicates (*n* = 3).

In general, a decreasing tendency in the content of soluble carbohydrates was observed in both leaves and roots of *S. biloba* after 10 days of exposure to 100 μM Cd (Figure 5). However, after 24 h into the experiment, only the leaves showed a significant decrease in the amount of soluble sugars in relation to the control.

In contrast, the content of soluble carbohydrates in the roots did not significantly (*p* < 0.05) decrease until the fifth day of the experiment (Figure 5). In addition to the damage of root cells, the increased bioaccumulation of Cd can induce the mobilization of sugar molecules where they are required to preserve the osmotic homeostasis of the root cells. Carbohydrate compounds not only act as structural cellular constituents of the cell walls, but also as intracellular signaling molecules involved in the regulation of metabolic processes associated with ATP production and cell energy management [52].

### 3.4. Further Analysis—Cd Bioaccumulation vs. Physiological Parameters

**Figure 6:**
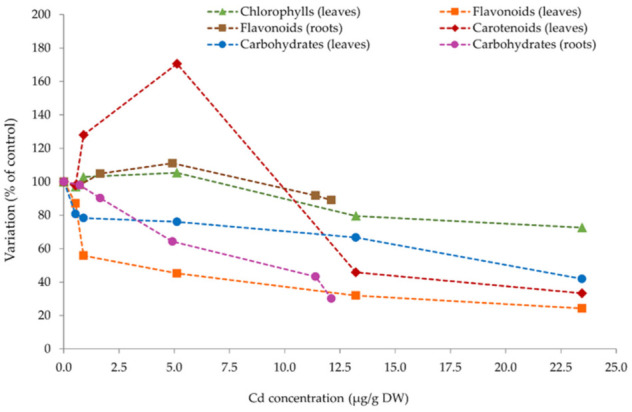
Variation of the assessed physiological parameters in relation to Cd bioaccumulation over time in the leaves and roots of *S. biloba*. The parameters chlorophylls (leaves), carotenoids (leaves), flavonoids (roots and leaves), and carbohydrates (roots and leaves) are presented as percentages of those obtained in the control plants. Data are reported as the mean value from three biological replicates (*n* = 3).

As displayed in Figure 6, the accumulation of Cd in the plant tissue of *S. biloba* influenced the assessed physiological parameters to different extents. At the end of the experiment, when maximum Cd bioaccumulation was registered, the parameters that exhibited greater discrepancy in relation to control were flavonoids (leaves) > carbohydrates (roots) > carotenoids > carbohydrates (leaves) > chlorophylls > flavonoids (roots). At top Cd concentrations in both plant organs, the reduction of flavonoids was greater in leaves (23.5% of control) than in roots (89.0% of control), whereas the carbohydrate decrease was higher in roots (30.2% of control) than in the leaves (41.9% of control). The spike in carotenoid contents when Cd levels in leaves were 5 µg/g (day 5 of the experiment, Figure 3), highlights the relevance of the results discussed in Section 3.3.1., i.e., the production of carotenoids as a response to the Cd-induced oxidative stress.

**Figure 7:**
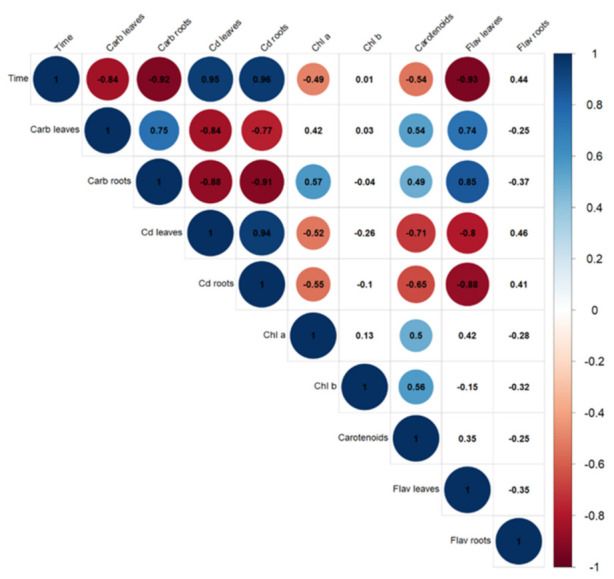
Pearson correlation coefficients between the assessed parameters. Correlations are significant at *p* < 0.05, except on uncolored cells. Colored cells denote a positive or negative correlation between parameters as per the heatmap, i.e., darker blue cells indicate a strong positive correlation (towards +1), while darker red cells represent strong negative correlations (towards −1). In addition, the specific correlation coefficients between parameters are presented in the corresponding cells. For instance, there is a strong negative correlation (dark red, −0.91) between soluble carbohydrate contents in the roots and Cd concentration in the roots.

The correlations depicted on Figure 7 further emphasize the relationship between Cd exposure time, its bioaccumulation in the roots and leaves of *S. biloba*, and the effect in the assessed physiological parameters. As expected, significant positive correlations were found between the experiment time and Cd concentrations in the roots and leaves (also positively correlated between each other). Conversely, both parameters showed significant negative correlations with the concentrations of soluble carbohydrates (roots and leaves), chlorophyll *a*, carotenoids, and flavonoids (leaves). These relationships are aligned with the results presented in the sections above and underline our discussion about the detrimental impact of Cd in *S. biloba* over time.

## 4. Conclusions

We have assessed the effect of Cd in naturally occurring *S. biloba* macrophytes growing in wetlands of the Middle Paraná River (Argentina). The accumulation of Cd in plant tissue induced evident visual alterations in plant leaves during 10-day metal exposure. Additionally, some of the assessed physiological parameters in *S. biloba* roots and leaves were more sensitive than others to the harmful effects of Cd bioaccumulation. Flavonoids in *S. biloba* leaves were the most affected parameter. On the other hand, chlorophyll levels were less impacted by Cd damage, while carotenoids showed a different time-dependent response. In addition, a gradual decrease in the content of soluble carbohydrates in both organs was observed over time. Therefore, the presented results demonstrate that *S. biloba* displays dissimilar physiological responses in plant roots and leaves to counteract the metal phytotoxicity. Additionally, the presented results highlight the significance to perform long-term metal exposure trials to identify additional physiological damage, thus becoming an important tool for the selection of tolerant macrophytes for biomonitoring purposes. Future research should explore a wider range of elements and their corresponding concentrations, to further inform about the potential use of this locally available species as a metal-pollution bioindicator.
